# Chip Integration: A Three-In-One Self-Powered NO_2_ Sensing System

**DOI:** 10.1021/acsomega.5c00086

**Published:** 2025-07-10

**Authors:** Zi-Fan He, Shafna Kunnathumpeedika, Iping Lee, Tzu-Chien Wei, Chi-Chang Hu

**Affiliations:** † Department of Chemical Engineering, 34881 National Tsing-Hua University, Hsinchu 300, Taiwan; ‡ Center for Emergent Functional Matter Science, National Yang Ming Chiao Tung University, Hsinchu 300093, Taiwan

## Abstract

Portable or miniaturized gadgets have seen rapid development in recent years, yet their power supply remains a major obstacle, often relying on external sources. Herein, we present a portable self-powered device for sensing the NO_2_ gas. This concept integrates a perovskite photovoltaic cell (8.84% conversion efficiency) for energy harvesting, a sodium-preintercalated δ-type MnO_2_-based supercapacitor (energy density of 0.76 μWh cm^–2^ at a power density of 0.025 mW cm^–2^) for energy storage, and a graphene nanoplatelet-based NO_2_ sensor (10.8% response at 10 ppm of NO_2_) as the energy consumption module, all on a single glass substrate in a miniaturized scale. Under illumination, the perovskite solar cell generates electricity, and the supercapacitor stores the energy and regulates the output voltage for powering the NO_2_ sensor. The high-level integration, achieved through rationally designed and modularized components, minimizes inactive spaces and eliminates cumbersome connections. This study introduces a modular and scalable platform for integrating energy harvesting, storage, and consumption on a single chip, essential for the next generation of ubiquitous electronic devices.

## Graphical abstract

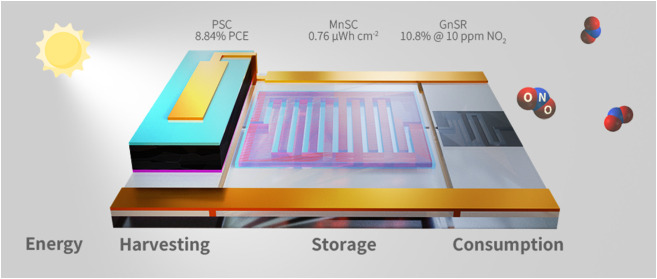

## 1 Introduction

Sensors have gained increasing attention for their role in real-time monitoring across diverse fields, including pharmaceuticals, agriculture, forensics, food safety, and environmental science.^1,2^ Among these, gas sensors, particularly for nitrogen dioxide (NO_2_), are critical due to the toxic effects of NO_2_ even at trace levels.^3^ Exposure to NO_2_ concentrations below 10 ppm can lead to symptoms, such as respiratory irritation, fatigue, and nausea.^4–12^ Since NO_2_ emissions are prevalent in both industrial and urban environments,^13,14^ the development of compact and cost-effective sensing systems is essential for public health monitoring.

Self-powered sensing systems offer a practical solution by enabling autonomous operation without the need for external power sources or frequent maintenance.^15–20^ These systems preferably harvest energy from natural sources such as solar, thermal, or mechanical energy. Among various energy sources, solar energy is particularly attractive due to its abundance and clean nature. However, its intermittent nature necessitates the integration of storage units, such as miniaturized supercapacitors or batteries, to stabilize output and maintain continuous sensor operation.^21–26^ Moreover, directly connecting photovoltaic devices to storage units has proven effective for omitting DC-DC converters and power tracking circuits, as their voltage ranges often overlap.^27^

The development of self-powered systems has progressed steadily over the years. Photopowered supercapacitors have garnered interest as they utilize the ubiquitous light source and leverage the high power density of supercapacitors to adapt the ever-changing solar energy.^28–30^ However, challenges such as material compatibility, electrolyte leakage, and the lack of standardized testing persist.^30^ Early designs typically connected solar cells to energy storage units using external wires or circuit boards.^31–33^ While these systems demonstrated basic functionality, their layouts were often bulky and rigid, making them unsuitable for miniaturized or portable applications. Later approaches adopted shared conductive substrates in either planar^34,35^ or stacked^36–48^ configurations. Planar structures offer the advantage of modularity and functional expandability, but they require further miniaturization to reduce the inactive space. Stacked devices can achieve energy density but often limit accessibility and reconfiguration. In addition, fiber-based systems with coaxial, twisted, or parallel configurations have also been explored for their mechanical flexibility in wearable applications.^49–52^ However, these structures generally suffer from low energy efficiencies and still lack seamless integration of sensing functions. These developments demonstrate the progress in integrating energy harvesting and storage, highlighting the need to incorporate an energy consumption component to realize practical, function-oriented applications.

Despite these advancements, the integration of energy consumption units, particularly sensors, into such systems has remained a challenge. Most reported devices rely on commercial solar cells and sensors connected via external cables or sockets, resulting in poor compatibility and restricted customization.^53–57^ The lack of design flexibility in these commercial components further limit optimal performance and arrangement.^58–63^ As a result, a fully integrated system combining energy harvesting, storage, and consumption on a single compact chip with a minimal inactive area has not yet been achieved.

Herein, we present a miniaturized, self-powered platform demonstrated through the development of a polarized NO_2_ sensing device. This 3-in-1 system exemplifies the integration of a perovskite solar cell for energy harvesting, a MnO_2_-based supercapacitor for energy storage, and a graphene nanoplatelet-based NO_2_ sensor for energy consumption. All three modules are fully integrated on a single planar substrate with minimal inactive spacing and a consistent voltage output. Fabrication techniques such as electrodeposition, spin-coating, spray-coating, and dip coating are optimized for compatibility with diverse materials, including perovskite, metal oxides, and carbon nanomaterials. Two working modes are used as the testing protocol for photopowered supercapacitors. This customizable, self-powered integrated system paves the way for a practical, personal self-powered electronic platform. To our knowledge, this is the first study to integrate a perovskite solar cell, a storage unit, and a gas sensor on a single glass substrate.

## 2 Experimental Section

### 2.1 Chemicals and Fabrication of Each Component

Fluorine-doped tin oxide (FTO) glass substrates (2.2 mm thick, 8 Ω sq^–1^) were patterned by using laser scribing, followed by sequential cleaning via ultrasonic treatment in 4% detergent solution and deionized water. After rinsing and drying, the substrates were treated with UV/ozone for 10 min to increase hydrophilicity.

For the perovskite solar cell (PSC), a compact TiO_2_ layer was deposited on the cleaned FTO via spray pyrolysis at 450 °C using a precursor solution of titanium diisopropoxide bis­(acetylacetonate). A mesoporous TiO_2_ layer was then spin-coated using a diluted TiO_2_ paste and sintered. The MAPbI_3_ perovskite film was deposited via a one-step spin-coating method using a DMF:DMSO precursor solution with chlorobenzene added as an antisolvent. After annealing, a methylammonium gas treatment was applied to enhance crystallinity, followed by the deposition of the spiro-OMeTAD hole transport layer and thermal evaporation of a gold electrode through a patterned mask. The MAPbI_3_ film shows a uniform thickness of approximately 460 nm, as determined from focused-ion beam microscopy cross-sectional images in our previous work.^64^

The Na_*x*_MnO_2_-based supercapacitor was fabricated via electrodeposition at 0.32 mA cm^–2^ for 1250 s from an aqueous solution containing 0.1 M Mn­(CH_3_COO)_2_ and 0.2 M Na_2_SO_4_, using a Pt-mesh counter electrode. The resulting electrodes were sealed with a drilled glass cover and UV-curable gel, and the electrolyte (0.1 M Na_2_SO_4_) was injected into the cavity before being sealed with Kapton tape.

Graphene gas sensors were prepared by dip-coating exfoliated poly­(vinylpyrrolidone) (PVP)-stabilized graphene sheets onto FTO. The graphene solution was prepared via 24 h sonication of graphite and PVP in water, followed by centrifugation. Substrates were surface-treated with a cationic surfactant before being dipped into the graphene suspension. After five dipping cycles and rinsing, the films were annealed at 325 °C to remove residual surfactant. The PVP-graphene nanoplatelets exhibit thicknesses ranging from 3.597 to 17.92 nm, corresponding to approximately 12 to 50 graphene layers based on the ideal graphite interlayer distance, as reported in our previous study.^65^

Further details of the chemical compositions, precursor preparation, and processing parameters are available in the Supporting Information.

### 2.2 Fabrication of Photo-Capacitors

The photocapacitor was fabricated by integrating perovskite solar cells and Na_*x*_MnO_2_ supercapacitors. First, Na_*x*_MnO_2_ was electrodeposited on a patterned FTO substrate. Next, the perovskite solar cell was fabricated using the same method described in the previous sections. It should be noted that the Na_*x*_MnO_2_ film was protected with Kapton and plastic wraps before any deposition process. Finally, the perovskite solar cell and supercapacitor were encapsulated simultaneously by using commercial UV-gels (Eversolar AB-313, Everlight Chemical Industrial Corporation) before the final electrolyte injection.

### 2.3 Fabrication of Self-Powered Integrated Sensing Devices

The fabrication procedure for the self-powered integrated sensing devices is illustrated in Figure 1, with a sequence designed to minimize damage to the as-prepared film. The FTO substrate was first cleaned and patterned using laser scribing. Second, Na_*x*_MnO_2_ was electrodeposited onto the patterned FTO substrate. Third, the electron-transporting layer of the perovskite solar cell (consisting of compact TiO_2_ and mesoporous TiO_2_) was sequentially deposited by spray-coating and spin-coating. Next, PVP-graphene was deposited by using a two-step dip coating. After that, the perovskite absorber and hole transport layer were spin-coated. Last, the gold was deposited by evaporation with the designed aperture mask before encapsulation with UV-gels.

**Figure 1:**
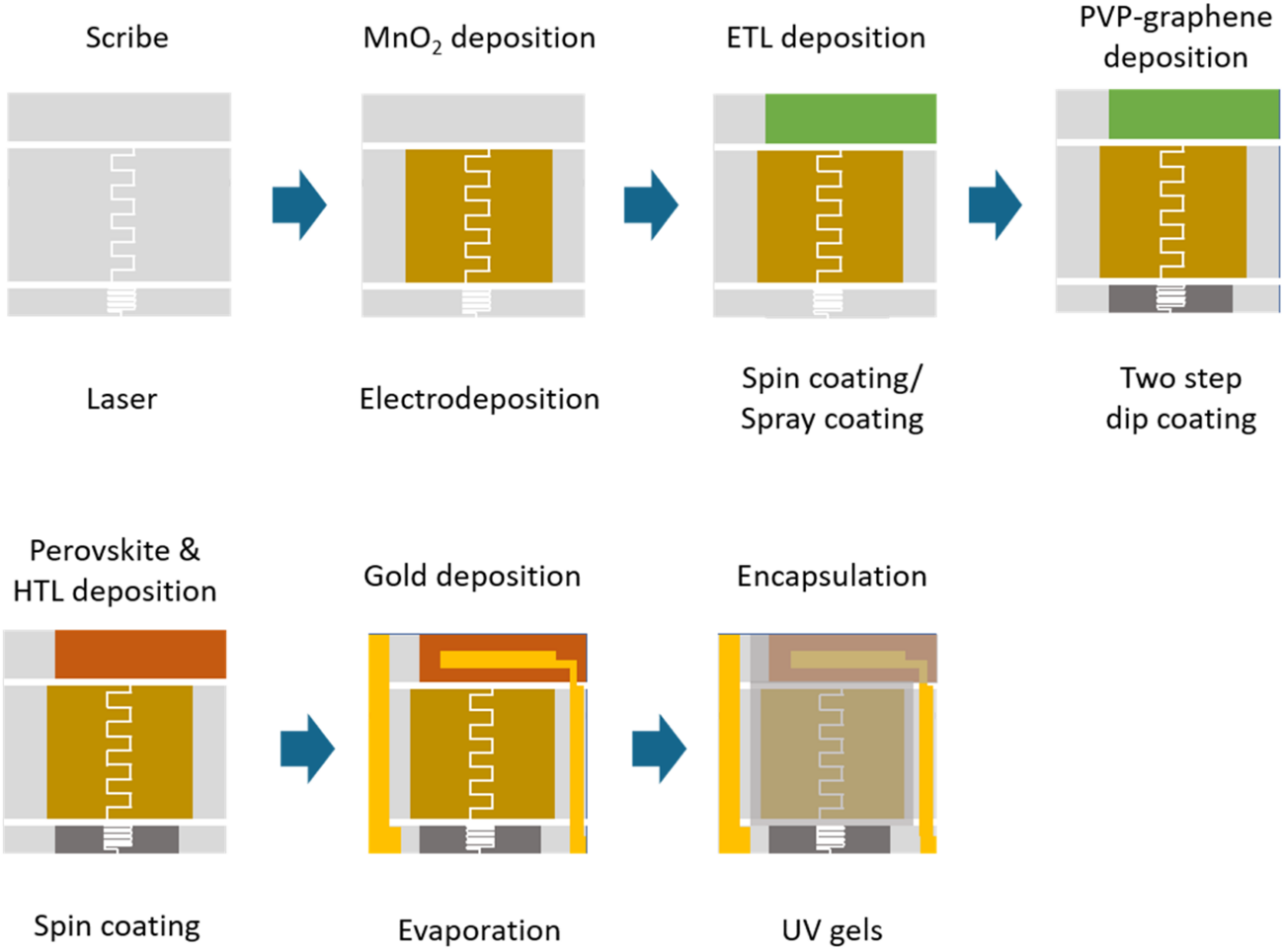
Fabrication procedure of the self-powered integrated sensing system.

## 3 Results and Discussion

### 3.1 Layout of Self-Powered Integrated Sensing Systems

The photograph and illustrations of the miniaturized 3-in-1 system are shown in Figure 2. The cross-sectional views of the perovskite solar cell (PSC), MnO_2_-based supercapacitor (MnSC), and graphene nanoplatelet NO_2_ sensor (GnSR) are also shown in Figure 2. All components share the same conductive glass substrate (fluorine-doped SnO_2_-coated glass, FTO glass) which is patterned and interconnected by laser scribing. The PSC (Figure 2B) is configured in an n-i-p structure by sequentially depositing a TiO_2_ electron-transporting layer, the MAPbI_3_ light-absorbing layer, a spiro-OMeTAD-based hole transporting layer, and an Au electrode onto the FTO substrate. A MnSC is arranged downstream to store the photogenerated energy from PSC and to regulate the voltage output for GnSR. The MnSC (Figure 2C) is constructed by two electrodeposited Na_*x*_MnO_2_ symmetrical electrodes and an aqueous Na_2_SO_4_ electrolyte.^66^ The GnSR (Figure 2D) is designed at the end of the circuitry with poly­(vinylpyrrolidone)-exfoliated graphene nanoplatelets (PVP-graphene) deposited onto an interdigitated electrode zone via a surfactant-assisted dipping process.^67^ Accordingly, the system operates by converting ambient light into electrical energy from the PSC, storing the photogenerated electricity in the MnSC, and then powering the GnSR.

**Figure 2:**
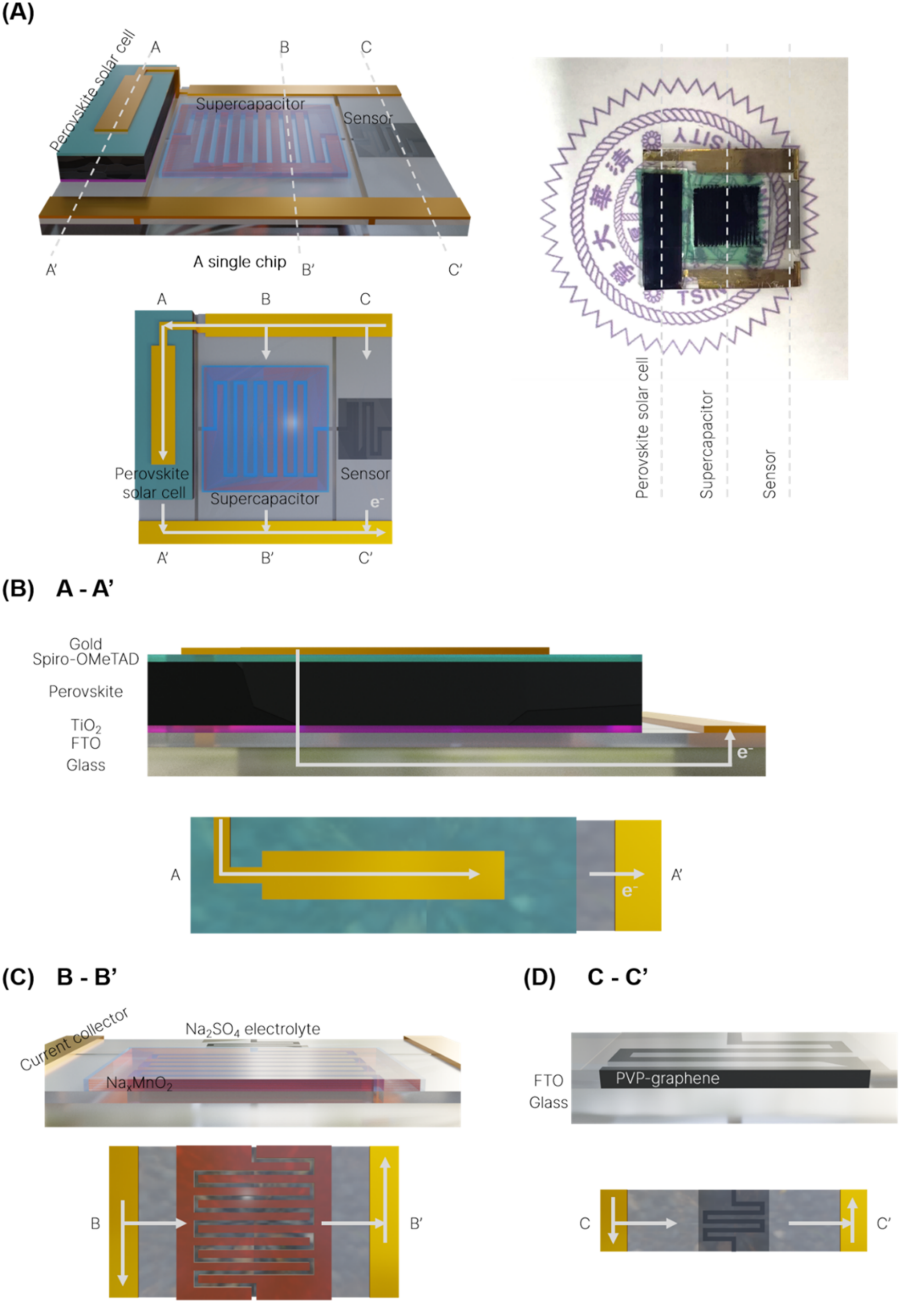
(A) Structural schematic and photograph of the self-powered sensing system integrating a perovskite solar cell, a supercapacitor, and a gas sensor on a single glass substrate. (B) A-A′ cross-sectional illustration of the perovskite solar cell. (C) B–B′ cross-sectional illustration of the MnO_2_ symmetric interdigitated supercapacitor. (D) C–C′ cross-sectional illustration of the PVP-graphene gas sensor.

### 3.2 Effects of Pattern Design and Performance of PSCs

Characterizations of the PSC are provided in the Supporting Information. Figure S1 shows the scanning electron microscope (SEM) topography and X-ray diffraction (XRD) pattern of the MAPbI_3_ film, demonstrating its intact and smooth surface with a high crystallinity. Specifically, two patterns (Pattern I and Pattern II) were investigated on the performance of the PSC (Figure 3). The primary difference between the two patterns is the electrical pathways. As indicated by the arrow signs in Figure 3A, FTO is the major conductive pathway in Pattern I, while the main conductive path is through thermally deposited gold in Pattern II. Figure 3B compares the power conversion efficiency (PCE), short-circuit current (*J*_SC_), open-circuit voltage (*V*_OC_), and fill factor (FF) of the control PSCs and patterned PSCs. Under the same fabrication condition, the control device (active area of 0.056 cm^2^) exhibits an averaged PCE of 17.31% (*J*_SC_ = 22.67 mA cm^–2^, *V*_OC_ = 1.01 and FF = 0.76). However, the integration of the PSC into the pattern results in a PCE reduction. The average photovoltaic parameters of PSCs fabricated on Pattern II (PCE = 8.84% ± 0.80%, *J*_SC_ = 20.14 ± 2.94 mA cm^–2^, *V*_OC_ = 1.02 ± 0.02 V, and FF = 0.44 ± 0.06) outperforms those of the ones fabricated on Pattern I (PCE = 2.64% ± 0.83%, *J*_SC_ = 11.52 ± 1.85 mA cm^–2^, *V*_OC_ = 0.94 ± 0.01 V, and FF = 0.25 ± 0.07), which is due to the improved control of internal resistance. Light intensity analysis of PSCs with two patterns (Figure S3) reveals that the series resistance dominates their PCEs when the light intensity decreases and FF increases dramatically. Notably, the PSC with Pattern II design surpasses that with Pattern I design in all investigated light intensities, leading us to choose Pattern II for further discussion. However, it is worth noting that the 3-in-1 system may be used in various light conditions, and the design of Pattern 1 may be adoptable for use in the dim light environment as its FF can increase to 0.72 under a low light intensity of 3.4 mW cm^–2^.

**Figure 3:**
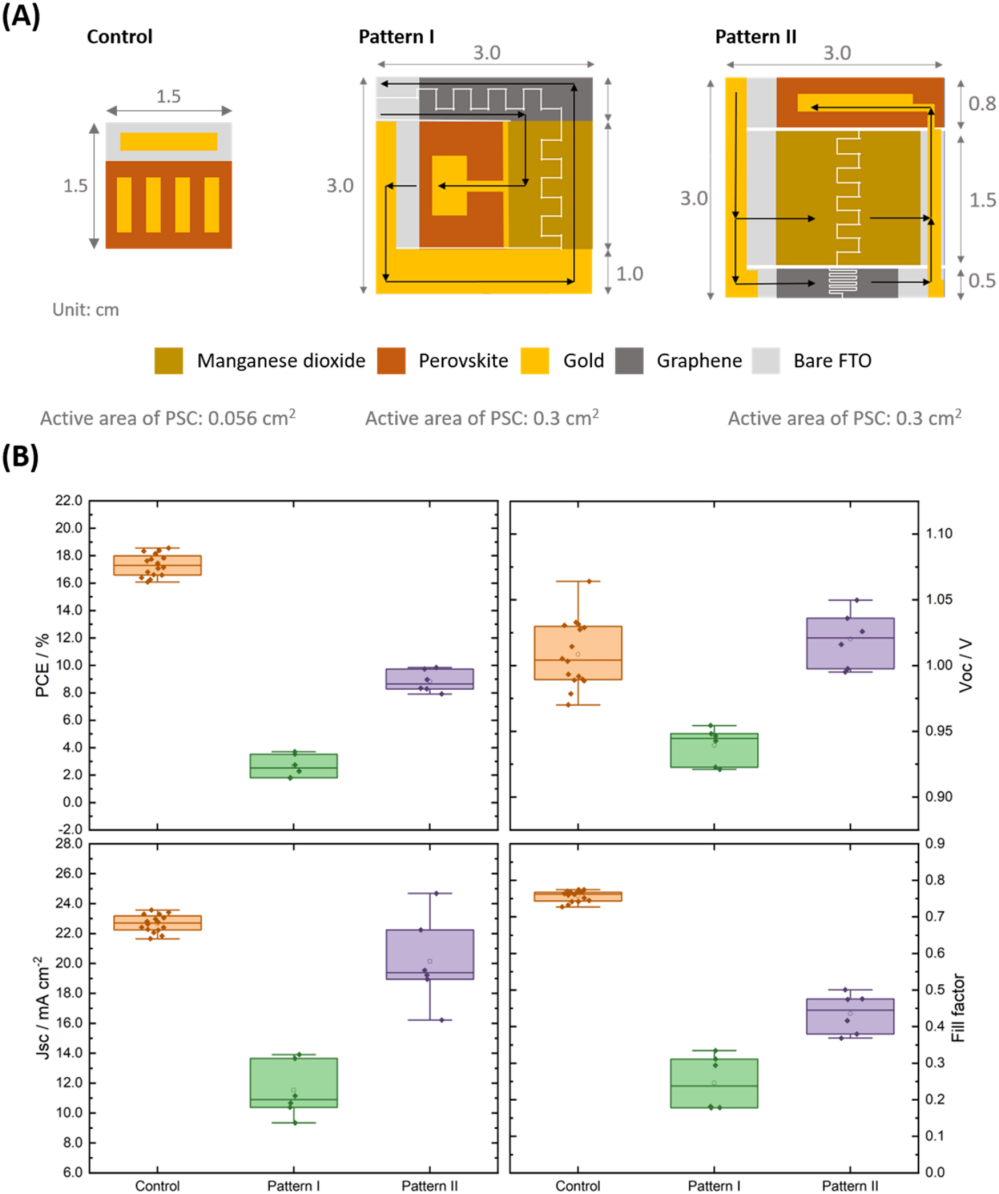
(A) Control cell and the two integration patterns of PSC devices. (B) Photovoltaic performance of PSCs based on the standard control design (active area = 0.056 cm^2^), Pattern I (0.3 cm^2^), and Pattern II (0.3 cm^2^).

### 3.3 Performance of Interdigitated MnSC

We have previously developed a sodium-preintercalated δ-type MnO_2_ (Na_*x*_MnO_2_) electrode with high specific capacitance, excellent capacitance retention, and high rate performance.^66^ These properties, along with its operating cell voltage (∼1.0 V), are well suited for storing the energy generated from upstream PSC. Here, we developed a symmetrical interdigitated MnSC, with characterization details provided in the Supporting Information. Figure S4 presents the configuration of interdigitated MnSC and Figure S5 shows the XRD pattern, X-ray photoelectron spectroscopic element survey spectrum, EQCM result of galvanostatic deposition, and surface morphology of manganese oxide preintercalated with cations. Figure 4A shows the cyclic voltammograms (CV) of the electrodeposited Na_*x*_MnO_2_ electrode in a three-electrode configuration, indicating the rectangular feature of ideal pseudocapacitive behavior. Moreover, the current response at 0.5 V is nearly linear to the scan rate which indicates the surface-controlled capacitive behavior. The high symmetry in CVs indicates the excellent reversibility, ascribed to the stable structure of Na_*x*_MnO_2_. In Figure 4B, the galvanostatic charge–discharge (GCD) curves of a symmetrical MnSC device at various current densities are presented. At current densities ≥0.2 mA cm^–2^, all GCD curves exhibit symmetric features with nearly perfect triangular shapes and negligible voltage drop, indicating the ideal charge/discharge behavior. The thickness of Na_*x*_MnO_2_ was optimized by adjusting the electrodeposition time, based on the linear relation between the thickness and deposition time shown in Figure S6. The result indicates that the thicker active material leads to higher capacitance, while the maximal operational electrodeposition time of Na_*x*_MnO_2_ is ca. 1250 s due to the adhesion concern, as observed from the optical microscopic images (Figure S7).^68–71^

**Figure 4:**
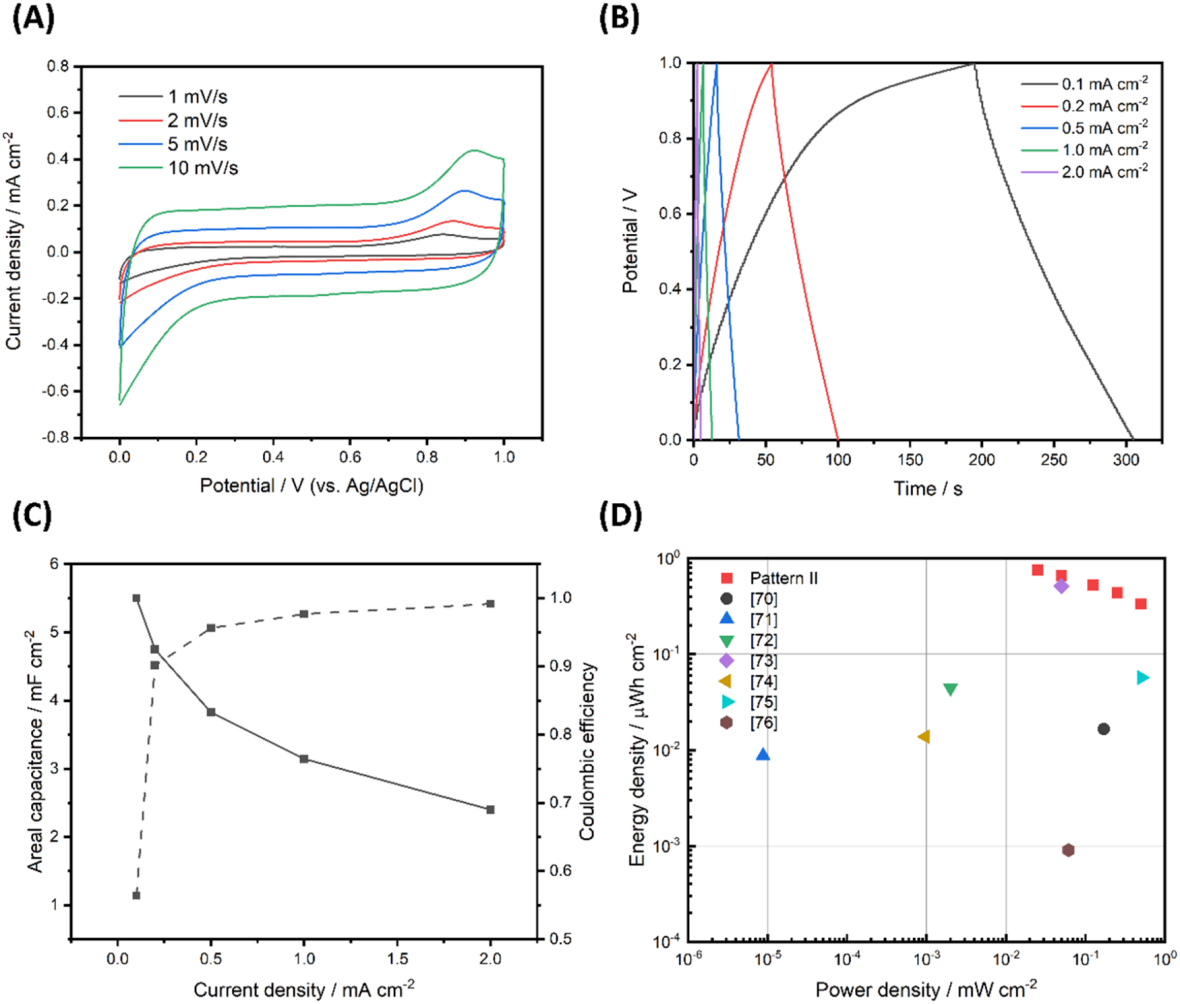
(A) Cyclic voltammograms of Na_*x*_MnO_2_ in a three-electrode configuration on Pattern II. (B) Galvanostatic charge–discharge curves of the Na_*x*_MnO_2_ symmetric supercapacitor at various current densities, and (C) their corresponding areal capacitance. (D) Ragone plot comparing the Na_*x*_MnO_2_ supercapacitor and other MnO_2_-based microsupercapacitors.

Based on the GCD curves, the specific capacitance (normalized by footprint area) dependence on the current density is calculated and displayed in Figure 4C. At a low discharge current density of 0.1 mA cm^–2^, the MnSC exhibits an imposing area capacitance of 5.5 mF cm^–2^. The current density and capacitance of the device are normalized by the footprint area of an electrode and the whole device, respectively (see Supporting Information Section 1.6). When the current density was increased to 2.0 mA cm^–2^, the capacitance retained 44% of its initial values, indicating an acceptable capacitance stability and rate capability for such a thin film configuration. A Ragone plot (Figure 4D) shows that our MnSC exhibits a maximum specific energy density of 0.76 μWh cm^–2^ at a power density of 0.025 mW cm^–2^, much higher than those reported for miniaturized MnO_2_-based supercapacitor.^72–78^ The achievement is attributable to sodium preintercalation on MnO_2_ and the electrodeposition for film preparation; the former increases specific capacitance of the energy storage materials, and the latter creates a uniform morphology without the need for binders.

### 3.4 Performance of GnSR

PVP-graphene as the sensing materials has been previously studied.^65^ Herein, the characterization of PVP-graphene-based GnSR is separately provided in the Supporting Information. Figure S8 depicts the configuration of GnSR, the surface topography of PVP-graphene, and the Raman spectrum of PVP-graphene. In principle, the conductivity of PVP-graphene changes upon exposure to NO_2_ gas, enabling the sensors to exhibit a current response at a fixed or fluctuating potential. Herein, we transplant the system to our 3-in-1 system as an example of the energy consumption component. Figure 5A shows the current response under 5 ppm of NO_2_ exposure at 0.5 V. The sensor exhibits a current response of approximately 5% after 250-s NO_2_ aeration (yellow sections). Moreover, the sensor can recover 87% of the total response by simply aerating air at room temperature. The relationship between the response of PVP-graphene sensor and the NO_2_ gas concentration are presented in Figure 5B. To ensure the complete recovery of the sensor, the sensor is heated at 200 °C for 1 h after every test. In the concentration range of 10–300 ppm, the response increased with increasing the NO_2_ concentration. In detail, these values after 1500 s of NO_2_ aeration were 10.8% (10 ppm), 16.4% (80 ppm), 20.1% (150 ppm), and 25.7% (300 ppm). As shown in Figure S9, the correlation between response and concentration is fairly linear with the R-squared of 0.9791, indicating PVP-graphene is a good NO_2_ sensing material up to 300 ppm of NO_2_ exposure.

**Figure 5:**
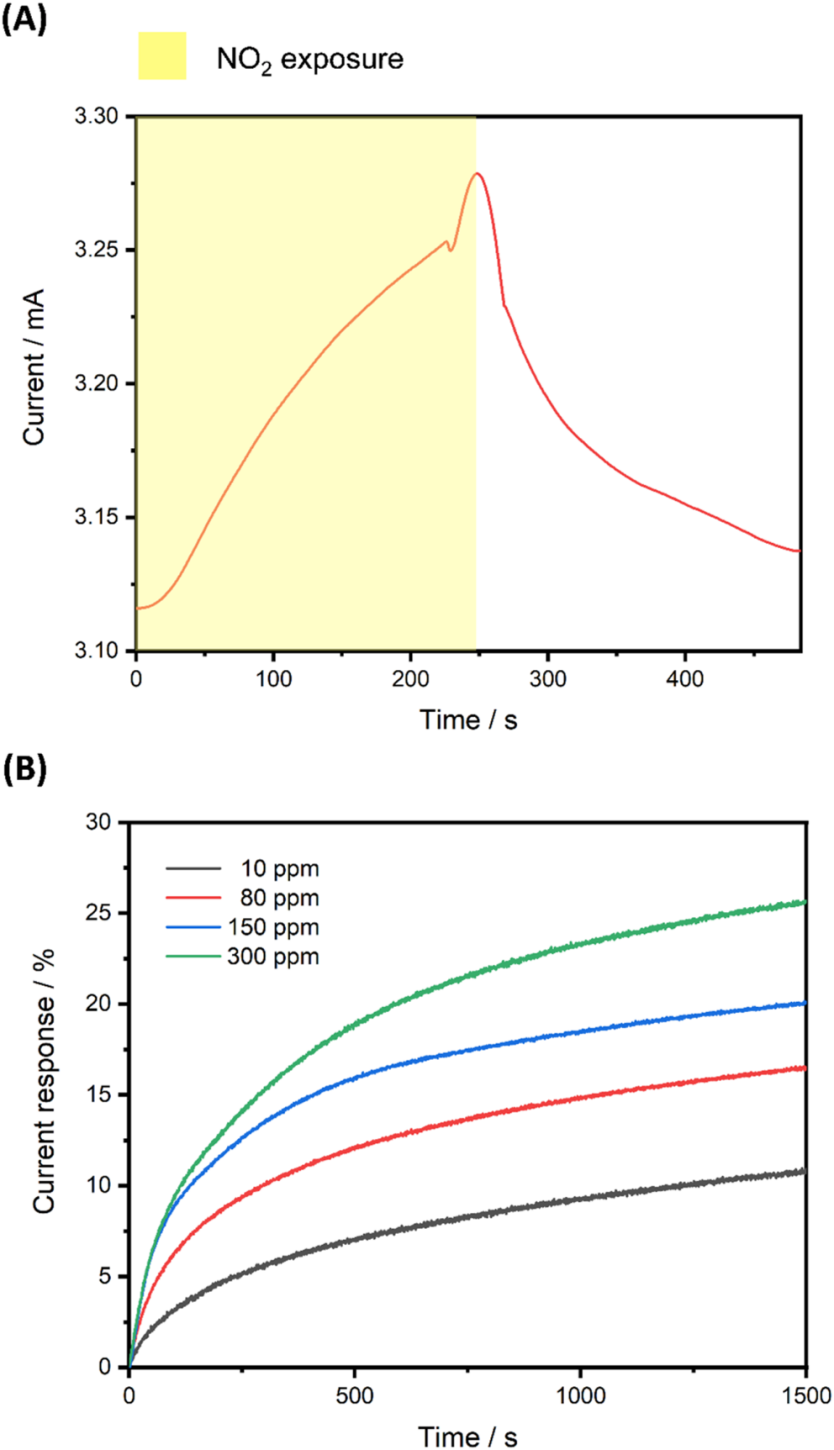
(A) Current response of the PVP-graphene sensor during the 5 ppm of NO_2_ gas test at 0.5 V. (B) Response curves at various NO_2_ concentrations.

### 3.5 Performance of Integrated PSC/MnSC Photocapacitor

The behavior of integrated photocapacitors consisting of a PSC and MnSC was studied before their use as a power source for gas sensors. Figure 6A displays the photocharging (zone 1) and dark galvanostatic-discharge (zone 2) curves of the photocapacitor at 0.1 mA cm^–2^ along with the corresponding current. The voltage of PSC and MnSC reach ca. 0.9 V within 22 s of photocharging, and after the 97 s of photocharging process under illumination, the voltage of PSC reaches about 1.0 V. Subsequently, the voltage and output current remain nearly constant for 200 s, indicating the good stability of the photocapacitor. The photocapacitor is then galvanostatically discharged in the dark, requiring 17.8 s to completely discharge. The calculated discharged capacitance is 1.8 mF cm^–2^, which is in the same order of magnitude of the individual MnSC discussed above. This capacitance loss may be attributed to the surface damage of Na_*x*_MnO_2_ during fabrication. Following the galvanostatic discharge, a reverse current occurs from the MnSC to the PSC (zone 3), which could cause significant problems for the shaded cell. However, these issues could be solved by incorporating the bypass diode after future commercialization.^79,80^ The behaviors of the photocapacitor discharged at various current densities and its efficiencies are further discussed in Figure S10.

**Figure 6:**
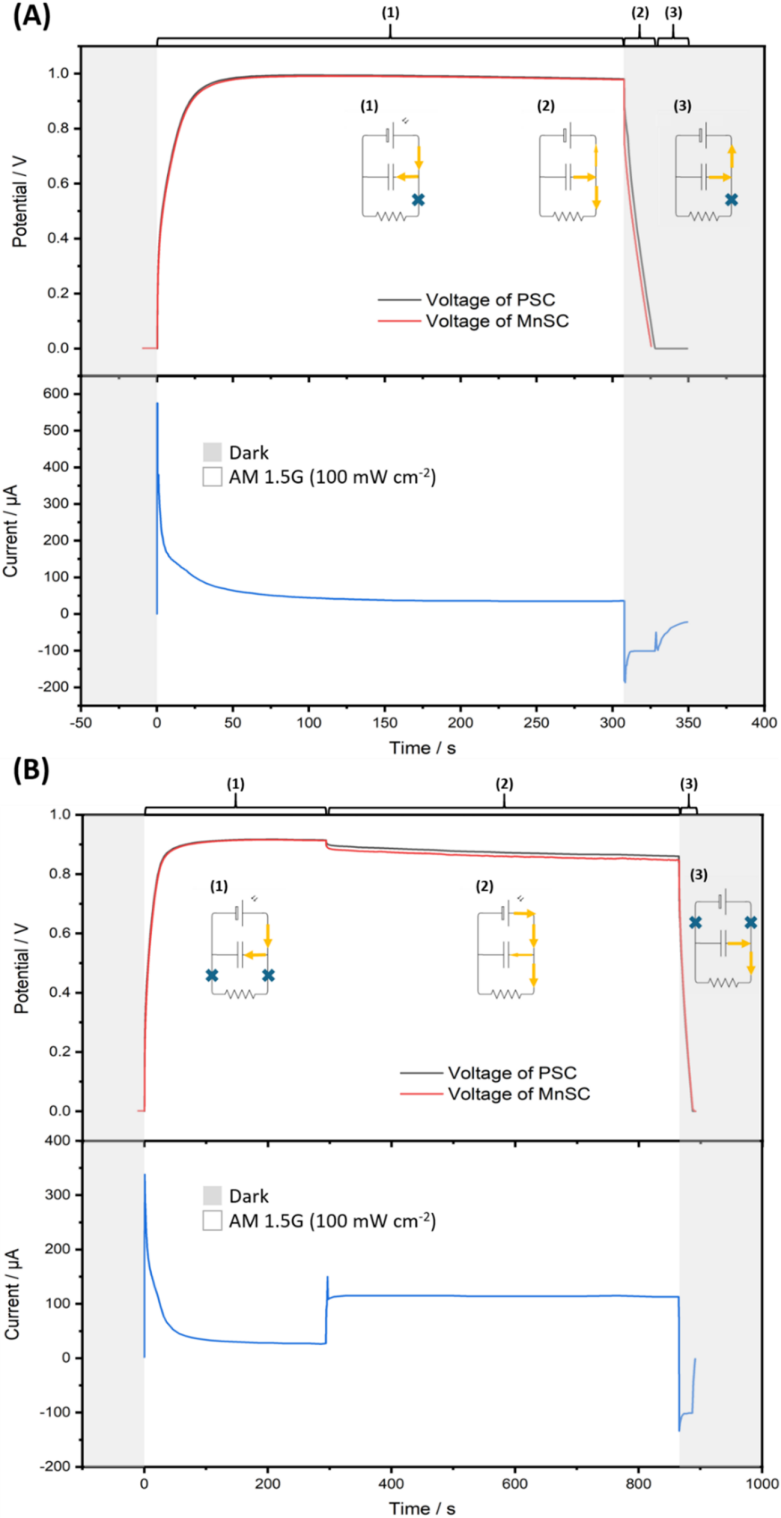
(A) Photocharging (white sections) and dark galvanostatic discharge (gray sections) curves of the photocapacitor at 0.1 mA cm^–2^. (B) Photocharging (white sections), light galvanostatic discharge (white sections), and dark galvanostatic discharge curves of the photocapacitor at 0.1 mA cm^–2^.

To simulate the practical use of the photocapacitor under light, Figure 6B displays the photocharging responses for a certain time (zone 1); then galvanostatic discharge under illumination (zone 2) and in the dark (zone 3). The photocapacitor shows similar charging behaviors in zone 1, followed by a voltage drop due to the forced galvanostatic current output in zone 2. In zone 2, the photocapacitor exhibits a steady voltage under loading, making it be a suitable power source for sensors that require continuous monitoring of NO_2_ levels over an extended period.

### 3.6 Performance of PSC-MnSC-GnSR Fully Integrated Sensing System

The cycling performance of photocharging and sensing was investigated. Figure 7A presents the voltage and current response with (yellow sections) and without (nonyellow sections) NO_2_ exposure. Under illumination, the integrated device is photocharged to 0.68 V in 250 s. Without the maximum power point tracking, the output voltage is close to the voltage of maximum power point derived from *J*–*V* curve in Figure S2 (0.58 V) due to the suitable power buffer of MnSC and sensor loading. When the device is exposed to 62.5 ppm of NO_2_, the GnSR exhibits a voltage response of 10.3% and the PSC voltage decreases to 0.61 V. The decreased voltage was attributable to the decreased resistance of the PVP-graphene sensor during NO_2_ sensing, in turn, increasing the current output of the PSC. Figure S11 also shows the stable power output of the solar cell in the system before and after NO_2_ exposure. The power density of 5.2 mW cm^–2^ is more than sufficient to power the sensing system, given the low power consumption of the fully charged supercapacitor (0.03 mW, Figure 6) and the gas sensor (0.16 mW, Figure 5). In the dark, the voltages of PSC and GnSR slowly drop to 0 V without the reverse bias, which benefits from the consecutive energy consumption from the sensor.

**Figure 7:**
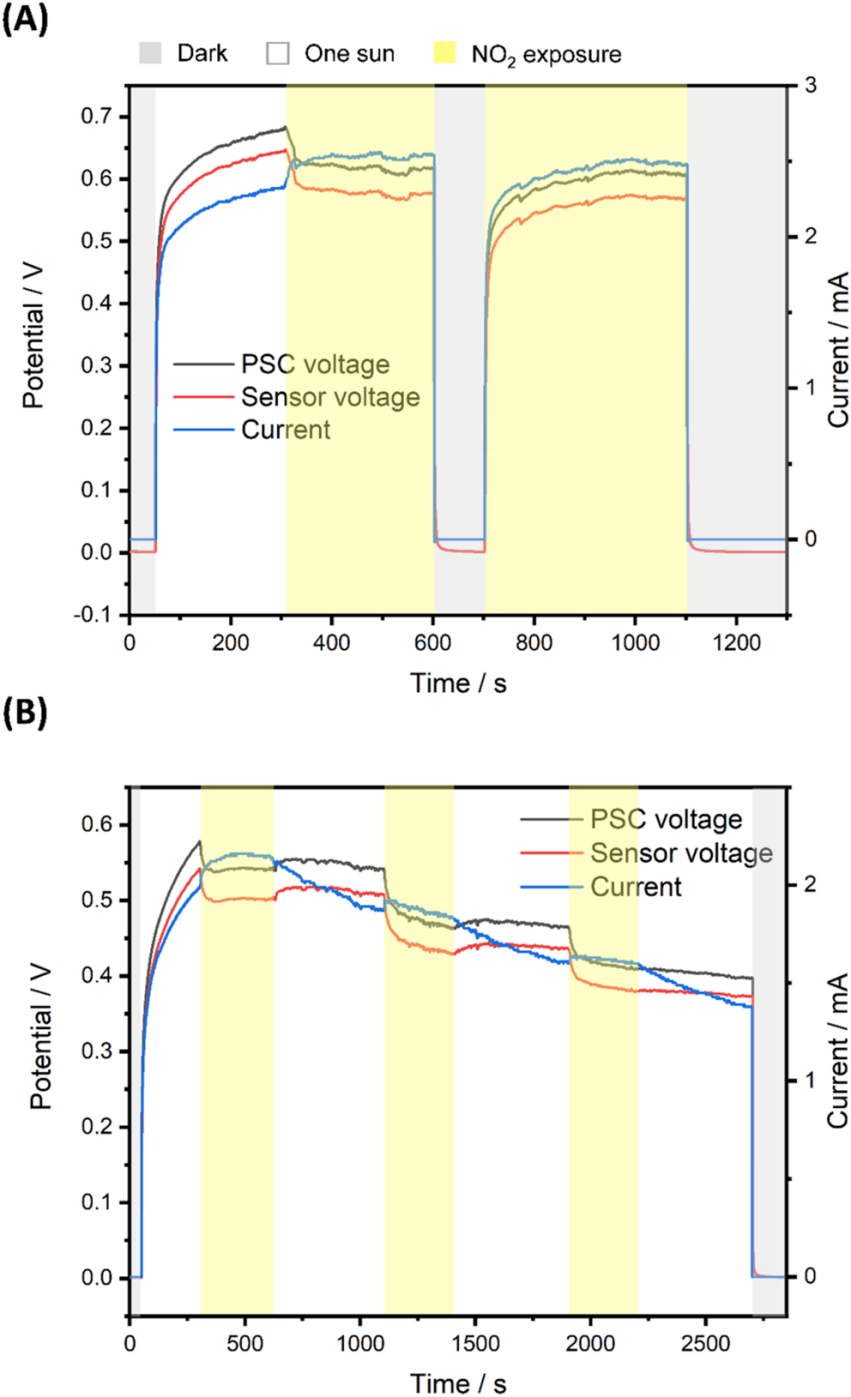
(A) Monitored potential and current response before and after NO_2_ exposure (uncolored sections) and after NO_2_ exposure (yellow sections). (B) Monitored potential and current response during a repeated NO_2_ sensing test.

In the second run, the voltage increased to the same voltage climax value of 0.61 V under identical NO_2_ conditions, indicating the steady sensing ability and the superior stabilizing capability from MnSC. Figure 7B shows the real-time responses of the integrated sensing device measured repeatedly without NO_2_ (uncolored sections) and with 62.5 ppm of NO_2_ (yellow sections). All three NO_2_ sensing runs showed a noticeable voltage drop in the PSC, with voltage responses of 6.9, 14.8, and 10.8%. For each sensor recovering cycle, the voltage of the PSC increased along with the decreasing current due to the increased resistance of the PVP-graphene sensor, indicating a good recovery behavior. Table S1 compares recent self-powered gas detection systems, manifesting the novelty and superiority of our device. Future studies will focus on exploring the long-term stability, humidity effects, selectivity analysis, and influence of interfacial structure and environmental conditions on integrated self-powered systems.^81–84^

## 4 Conclusions

The concept of a self-powered gas sensing system, including energy harvesting, storage, and consumption components, is realized through the rational design and integration of a MAPbI_3_-based perovskite solar cell, a Na_*x*_MnO_2_-based supercapacitor, and a graphene-based NO_2_ sensor. This self-powered system can sense the NO_2_ with timely and consistent voltage variation as long as there is light. This integrated platform allows design flexibility in the choice of electrode materials, substrate layout, and power management, enabling adaptation to various sensing targets or operating environments. Future exploration of this modularized platform can involve improving the PCE of the PSC, increasing the areal capacitance of the supercapacitor for additional electrochemical or electrical applications such as desalination, water splitting, and more.

## Supplementary Material



## Notes

Experimental procedures and characterizations for a three-in-one self-powered NO_2_ sensing system, including materials, fabrication of perovskite solar cells (Figures S1–S3), Na_*x*_MnO_2_ supercapacitors (Figures S4–S7), and PVP-Graphene sensors (Figures S8–S9); photocapacitor integration and efficiency (Figure S10); system power output (Figure S11); sensing performance comparison (Table S1) (PDF)
