# Supplementary material for: Chip Integration: A Three-In-One Self-Powered NO2 Sensing System
Source: ACS Omega. 2025 Jul 10;10(28):30116–26. doi: 10.1021/acsomega.5c00086 (PMC12290958; doi:10.1021/acsomega.5c00086)
Supplement: Supplementary file 1 [file ao5c00086_si_001.pdf]

Supporting Information

Chip Integration: A Three-In-One Self-powered NO_2 Sensing System

Zi-Fan He[1], Shafna Kunnathum Peedika[1], Iping Lee[1], Tzu-Chien Wei*[,1,2], Chi-Chang Hu*[,1]

[1]*Department of Chemical Engineering, National Tsing-Hua University, Hsinchu 300, Taiwan*
[2]*Center for Emergent Functional Matter ScieNnce, National Yang Ming Chiao Tung University, Hsinchu 300093, Taiwan*

*Corresponding author: Chi-Chang Hu, NTHU Chair Professor

Department of Chemical Engineering,

National Tsing Hua University,

Hsin-Chu, 300044, Taiwan

*Corresponding author: Tzu-Chien Wei, NTHU Professor

Department of Chemical Engineering,

National Tsing Hua University,

Hsin-Chu, 300044, Taiwan

The supporting information includes **11 Figures and 1 Table**.

Experimental details

1.1 Chemicals

Mn(II) acetate tetrahydrate (99.99%), acetic acid (Reagent plus, > 99%), sodium acetate, manganese(II) sulfate, sodium sulfate, sulfuric acid, zinc sulfate, sodium hydroxide were purchased from Sigma-Aldrich. Fluorine-doped tin oxide (FTO) electrodes (FTO, 2.2 mm-thick, 8 Ω sq^{-1}, Dyesol) were provided by Greatcell Energy.

1.2 Pattern Scribing and Pattern Cleaning

The fluoride-doped tin oxide glass was firstly patterned by laser scribing. Next, the FTO substrates were sequentially ultrasonicated in the solution of 4% commercial detergent (PK-LCG46, Parker International Co. Ltd., Taiwan) and reverse osmosis water for 30 min. After sonication, the FTO glass was rinsed with deionized water (DI water) and dried with an air blow gun. The clean substrate was finally placed in the UV/ozone machine for 10 min to remove any solvent and increase the hydrophilicity.

1.3 Fabrication of Perovskite solar cells

For the patterned substrates, there are definitive areas of perovskite solar cells and other components. Selective areas were practically protected by Kapton and plastic wraps before any deposition process.

The compact layer TiO_2 was deposited on a cleaned FTO substrate by spray pyrolysis at 450 ºC with a precursor solution of titanium diisopropoxide bis(acetylacetonate) and oxygen gas. The precursor solution was prepared by taking 75 wt.% diisopropoxide bis(acetylacetonate) in isopropanol, diluting with ethanol with a volume ratio of 1:9, and addition of 4 vol% acetylacetone. Next, the mesoporous TiO_2 was spin-coated onto the substrates via a commercial TiO_2 paste (30NR-D, Greatcell Solar Materials, diluted with pure ethanol at a ratio of 1:7 (30NR-D: ethanol in weight) at 6000 rpm for 30 s. After drying at 120 ºC for 10 min, the TiO_2 films were gradually sintered to 450 ºC and cooling to room temperature. The as-prepared TiO_2 substrate

was lastly treated by UV-ozone, and then transferred to humidity-controlled room (15% humidity) before perovskite deposition.

The perovskite layer was deposited onto TiO_2-coated substrates by one-step spin-coating. Firstly, the 1.5 M PbI_2 (99.0%, Xian Polymer Chemicals) 1.5 M MAI (home-made) and 0.35 M MACl (99.9%, GreatcellSolar) in a DMF:DMSO (99.9%, Sigma-Aldrich) mixed solvent (volume ratio = 8:2) was continuously spin-coated at 1000 rpm for 10 s and at 6000 rpm for 20 s. During the spin coating process, the chlorobenzene was dropped at the 16th second. Afterward, the prepared substrate was annealed at 120 ºC for 20 min for the formation of perovskite and cooled to room temperature before the methylammonium gas treatment.

The methylammonium (MA) gas treatment was performed in the glove box to avoid the moisture. In detail, the MA mixed solution was prepared by diluting the 33% methylamine ethanol solution into diethyl ether at the volumetric ratio of 1:4 in the glove box. The MA solution in the 50-mL recipient was then covered with the faced-down perovskite coated substrate for 3 s. Afterward, the treated perovskite samples were annealed at 100 ºC for 10 min for the sake of recrystallization and solvent removal.

A hole transport material solution containing 75 mM spiro-OMeTAD (>99.5%, Xi'an Polymer Light Technology Corp.) in chlorobenzene with 40 mM Li-TFSI (99.95%, Sigma-Aldrich) and 270 mM tBP (96%, Sigma-Aldrich) additives was spin-coated onto the substrate at 4000 rpm for 30 s.

Finally, a gold electrode was thermally evaporated onto the layer at the pressure of 1×10^{-6} millibar. For the patterned solar cell, the designed gold mask was stuck on the samples before gold evaporation to accurately limit the deposition area.

1.4 Characterizations of Perovskite solar cells

All the measurements were carried in ambient conditions (temperature: 25 ℃, relative humidity: 30%~40%) without any preconditioning. The I-V characteristics of

PSC was measured by a digital source meter (Keithley 2400, USA) under 1 sun, AM 1.5G simulated sunlight (Peccell Technologies, PEC-L15, Japan). A reference monocrystalline silicon photodiode (Oriel, USA) was used to calibrate the light intensity, and various photomasks was attached to the front side of the PSC to control the active area for control cells and patterned cells in **Fig. 3A**. Additionally, the light intensity-dependent analysis (**Fig. S3**) was done by using filters.

1.5 Fabrication of Na_xMnO_2 Supercapacitors

Na_xMnO_2 thin films electrodes on the patterned FTO substrate were obtained from electrodeposition at a fixed current density of 0.32 mA cm^{-2} for 1250 s in a two-electrode cell configuration, using an Autolab PGSTAT204 (Metrohm Autolab), and a Pt-mesh counter electrode. The electrodeposition was performed at ambient temperature in agitated aqueous solutions containing 0.1 M $Mn(CH_3CO_2)_2$ and 0.2 M Na_2SO_4.

The Na_xMnO_2 supercapacitor was encapsulated by using UV gels. In detail, the drilled glass was placed on the selective area with the UV gel around the edge. Next, the sample was exposed to UV light for 2 min in a vacuum. The electrolyte (0.1 M Na_2SO_4) was then injected into the gap between the substrate and the cover glass. An interdigitated supercapacitor was done by sealing the holes of the cover glass with Kapton.

1.6 Electrochemical measurement of Na_xMnO_2 Supercapacitors

The electrochemical properties were measured by CH Instrument in two-electrode configuration. The current density (i), areal capacitance (C_d), energy density (E_d), and power density (P_d) of device was obtained based on the following equation

$$i = \frac{2*I}{A_{fp}} \qquad \text{(Eq. 1)}$$

$$C_d = \frac{I*t}{A_{fp}*V} \qquad \text{(Eq. 2)}$$

$$E_d = 0.5 * C_d * \frac{V^2}{3600} \qquad \text{(Eq. 3)}$$

$$P_d = \frac{3600 * E_d}{t} \qquad \text{(Eq. 4)}$$

where I refers to discharging current, t denotes discharging time from galvanostatic charge-discharge curves, A_{fp} denotes the footprint area of the supercapacitor device, and V denotes voltage window.

1.7 Fabrication of PVP-Graphene Sensors

The preparation of PVP-graphene follows our past work.[1] To prepare the PVP-graphene solution, the graphite powder (0.1 g; 300 mesh, Sigma–Aldrich) and PVP (0.4 g; M_w=8000, Alfa Aesar) in 100 mL deionized water was stirred for 30 min at room temperature. The solution was subsequently sonicated for 24 h with a 300 W bath sonicator (DH-150, Delta) to exfoliate the graphite. After the sonication, the resultant solution was centrifuged at 500 rpm for 30 min to remove the large clusters. Next, the PVP-graphene was deposited onto the patterned FTO substrate by two-step dip-coating. In the first step, the FTO substrates was immersed into a 2% cationic surfactant (ML 371, OM Group) aqueous solution at 70°C for 10 min for surface modification. Subsequently, the surface-modified FTO substrate was dipped in the PVP-graphene aqueous suspension at room temperature for 10 min. Between each dipping process, the substrates were rinsed with deionized water to remove solvent and weakly adsorbed particles. After five-times two-step dipping process, the PVP-graphene FTO was heated at 325 ºC for 10 min to burn out the surfactant.

Characterization of Each Component

1. Perovskite solar cell

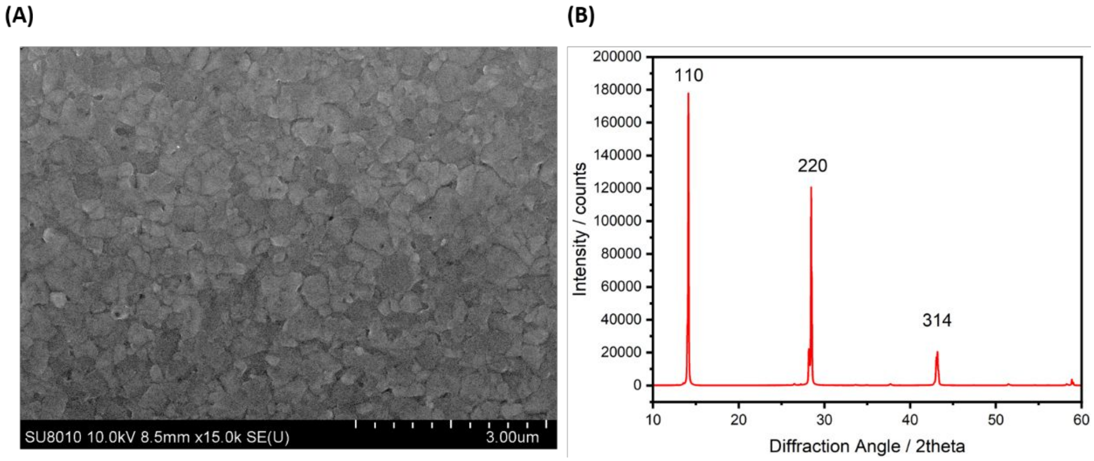

Figure S1. (A) The SEM image and XRD pattern of perovskite film on Pattern II after MA treatment.

Methylammonium (MA) gas treatment is introduced to improve the quality of the perovskite film. This treatment involves the interaction of the MA gas with the perovskite, resulting in the formation of a liquid intermediate that can undergo recrystallization under degassing and annealing. As shown in **Fig. S1**, the SEM image and XRD pattern demonstrate that the perovskite film treated with MA gas on Pattern II exhibits a smooth, pinhole-free surface, and the high purity and crystallinity.

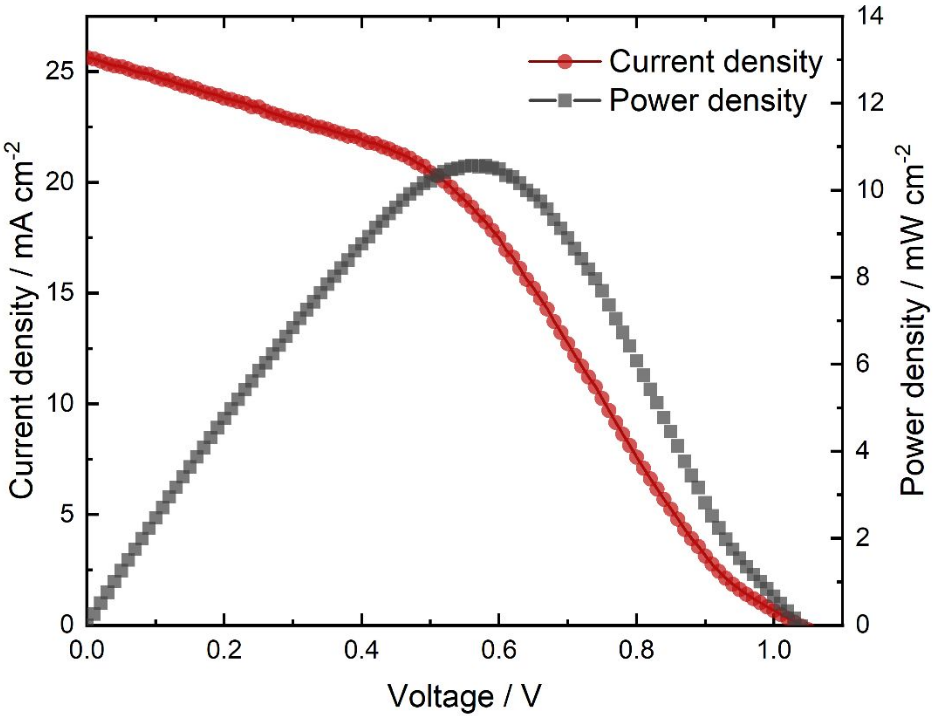

Figure S2. JV curve of the champion perovskite solar cell with Pattern II.

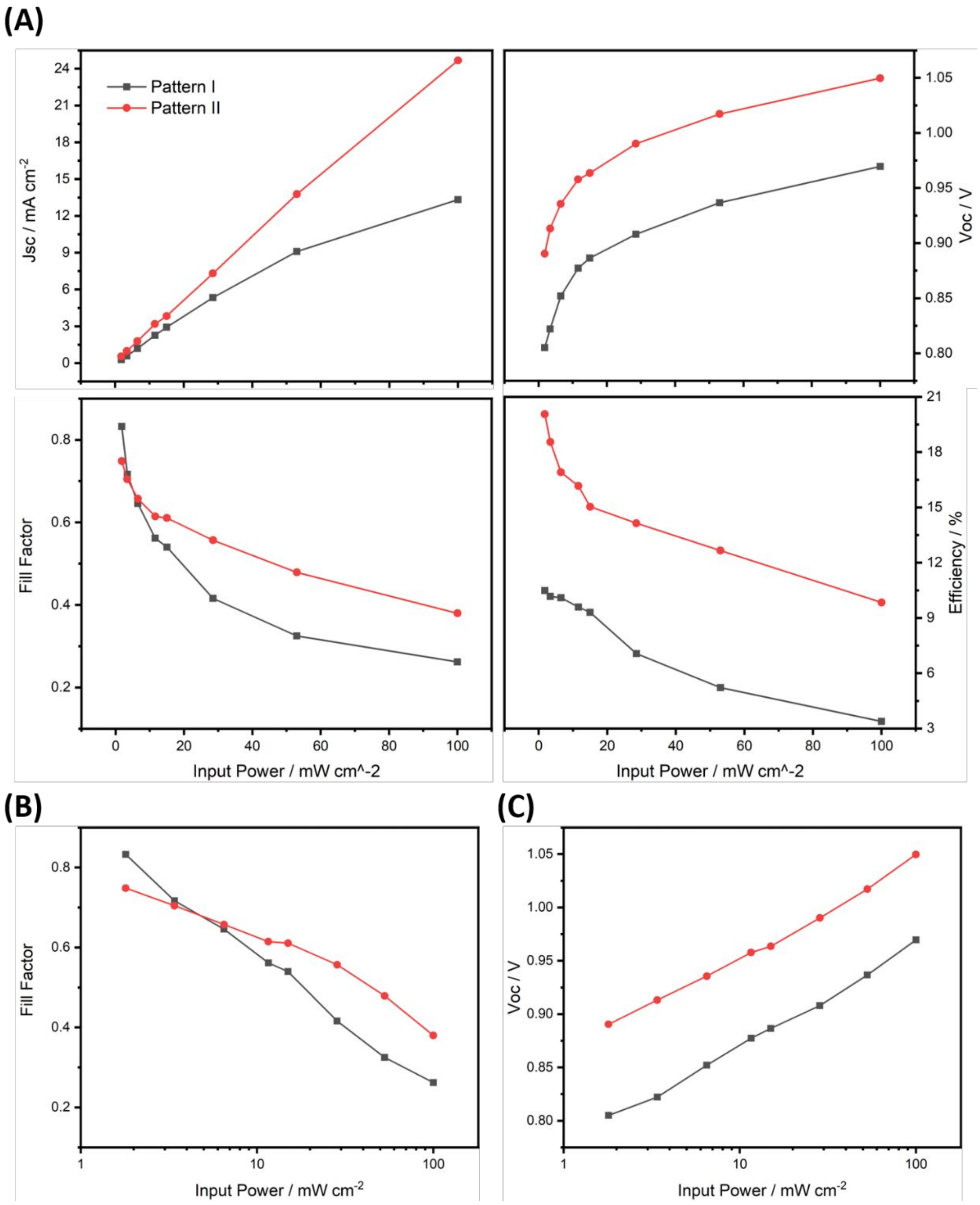

Figure S3. (A) Photovoltaic parameters of PSCs with Pattern I and Pattern II at various light illumination intensity. (B) V_{oc} and (C) FF as a function of logarithm of light intensity, respectively.

The light-dependent analysis was employed to investigate the resistance effect in perovskite solar cells with Pattern I and Pattern II. **Figure S3** shows the short-circuit

current, open-circuit potential, FF, and efficiency as functions of light intensity. The short-circuit current and open-circuit potential are decreasing as the light intensity decreases. The decrease in current results from the reduction in photo-generated current which is directly related to the carrier density. In addition, the V_{oc} decreases according to the equation $V_{oc} = \frac{nkT}{q} \ln \left(\frac{I_{ph}}{I_o} + 1\right)$, where n is the ideality factor, k is the Boltzmann's constant, and T is the cell temperature, q is the elementary charge, I_0 is the saturation current of the internal diode, and I_{ph} is the photo-generated current. On the other hand, the FF and efficiency increase and attain the maximum at the lowest intensity that can be prepared in the lab. This suggests that there are some effects limiting the perovskite under strong light. The maximal FF is 0.83 for Pattern I and 0.75 for Pattern II, which are near the Shockley-Queisser limit, indicating low recombination loss under dim light. The FF and V_{oc} are further plotted as the function of the logarithm of light intensity in **Fig. S3B** and **Fig. S3C** to extract the shape of FF curves and ideality factor. Compared to Pattern II, the FF curves in Pattern I exhibits the steeper change and more serious distortion, implying Pattern I suffer from server series resistance or recombination issue. The ideality factor is 1.59 for Pattern I and 1.51 for Pattern II, indicating the distributed SRH recombination via the bulk traps in solar cells with both patterns. The value coincides with the frequently reported value of 1.6, supporting the low or moderate recombination in the device. Therefore, the series resistance (instead of recombination) is found to dominate the solar cell performance under one sun, especially for the solar cells in Pattern I.

2. Na_xMnO_2 supercapacitor

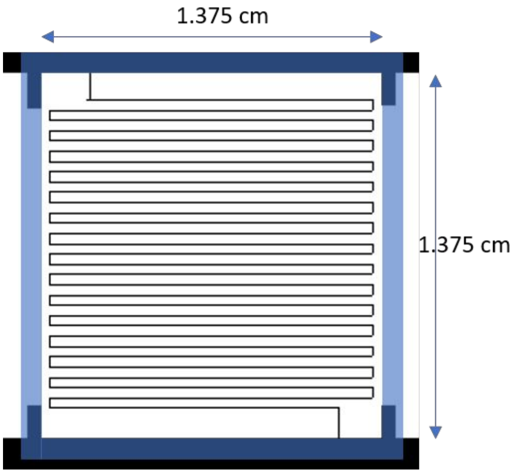

Number of fingers	30
Width of one finger	0.3575 cm
Length of one finger	13 mm
Interspace	22 μm
Active area	1.7992 cm^2
Footprint area	1.8906 cm^2

Figure S4. Configuration of the supercapacitor based on Pattern II.

Fig. S4 shows the geometry of the supercapacitors on Pattern II. The blue area is the dead area for encapsulation. The device area is calculated as the footprint area for the normalization of the capacitance test, while the MnO_2 deposited area (i.e. the footprint area minus the etched area), denoted as the active area, is used to calculate the current density during electrodeposition.

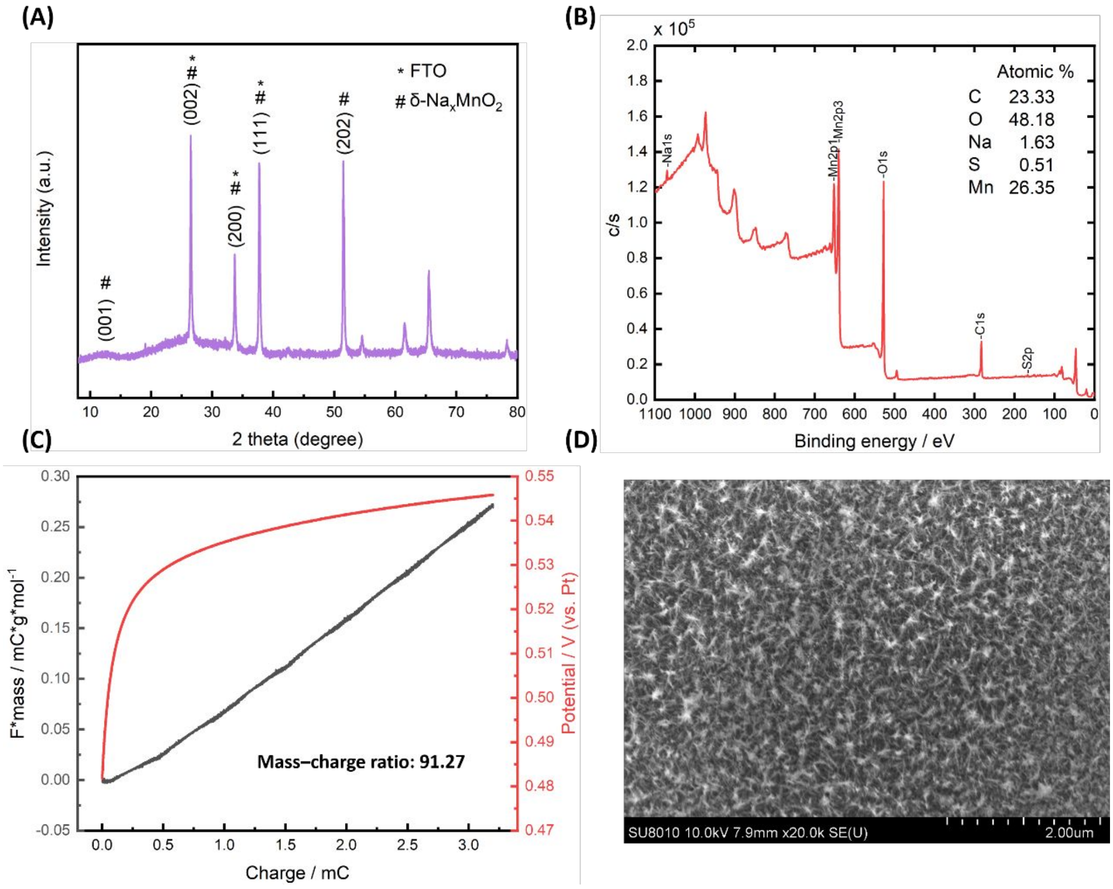

Figure S5 (A) X-ray diffraction pattern of electrodeposited manganese oxide. (B) X-ray photoelectron spectroscopic element survey spectrum of the manganese oxide deposit. (C) The EQCM result of galvanostatic deposition at a current density of 0.32 mA cm^{-2} for preparing Na_xMnO_2. (D) A scanning electron microscopic image of Na_xMnO_2.

Fig. S5A shows the X-ray diffraction (XRD) pattern of the electrodeposited manganese oxide complex. Sharp peaks at 26.4°, 33.7°, and 37.3° are identified to the facet (002), (200), (111) of δ-type Na_xMnO_2 (PDF# 43-1456).166-169 The δ-type Na_xMnO_2 (birnessite) is composed of layered edge-sharing MnO octahedra with an interlayer of hydrated cations. These pre-intercalated cations, such as K^+, Na^+, and Zn^{2+}, are thought to be a pillar to stabilize the structure and expand the interlayer spacing for better cyclability and faster ion transport. In the sample, the strong peak at 26.4° (002)

demonstrates the ordered and well-distributed sodium ions and crystal water in the interlayer of birnessite. The XPS result shown in **Fig. S5B** further validates the existence of sodium. With the average ratio of manganese and sodium of 0.16 obtained by using ICP-OES, the Na_xMnO_2 is denoted as $Na_{0.16}MnO_2$ for simplicity. The deposition of the reaction route is revealed by EQCM which records the mass change during galvanostatic deposition at the current density of 0.32 mA cm^{-2} (**Fig. S5C**). The formation of MnOOH dominates the deposition process with the mass-charge ratio of ca. 91 g mol^{-1}, matching the theoretical mass-charge ratio of ca. 87 g mol^{-1}. The extra mass is attributed to the incorporated ions and crystal water during the electrolysis. The proposed deposition reactions are listed in the following equations.

$Mn^{2+} - e^- + 2H_2O \leftrightarrow MnOOH + 3H^+$ (Eq. S1)

$MnOOH + xNa^+ + (x+1)e^- \leftrightarrow Na_xMnOOH + OH^-$ (Eq. S2)

$Na_xMnOOH \leftrightarrow Na_xMnO_2 + H^+ + e^-$ (Eq. S3)

The morphology and structure of Na_xMnO_2 were investigated by SEM in **Fig. S5D**. The ample wrinkles and folds are observed in Na_xMnO_2, enlarging the surface area. Moreover, the well-distributed pores with a diameter of about 70 nm provide ion channels to facilitate ion transport and diffusion. Overall, the Na_xMnO_2 with stabilizing pillars and uniform pores are expected to show excellent electrochemical performance as a supercapacitor electrode material.

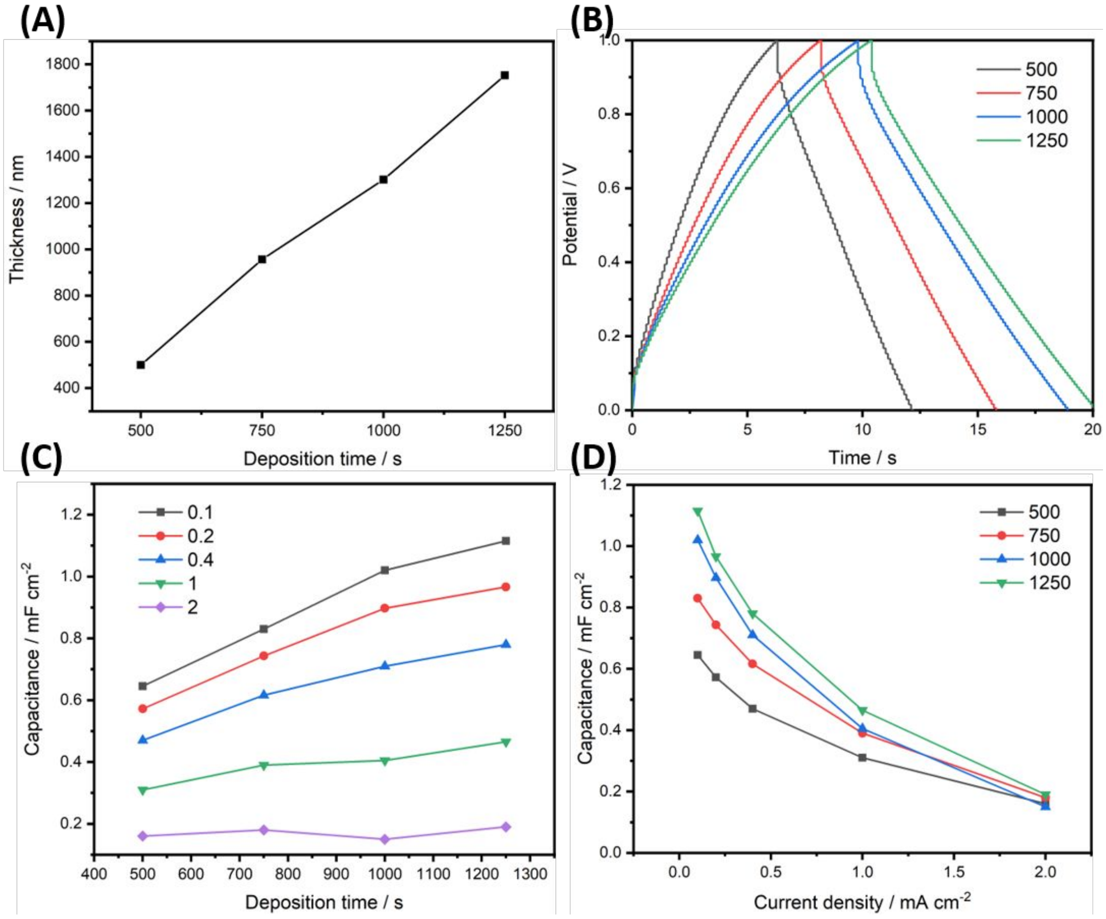

Figure S6 (A) Thickness and (B) charge/discharge curves of $Na_{0.16}MnO_2$ with four deposition times. The capacitance (C) as a function of deposition time and (D) as a function of charge/discharge current density.

Fig. S6 shows the effect of deposition time on the electrochemical performance of the Na_xMnO_2 symmetric supercapacitor. As expected, the deposition time is directly proportional to the thickness of active materials in the fixed active area (see **Fig. S6A**). The thicker Na_xMnO_2 is, the higher charge storage capacity is provided during charging/discharging. The chronopotentiograms of supercapacitors with four deposition conditions at 0.1 mA cm^{-2} are shown in **Fig. S6B.** Derived from chronopotentiograms, the capacitance of each group and their correlation between deposition time and current density can be retrieved (**Fig. S6C**). At low current densities,

the areal capacitance nearly linearly increases with the deposition time due to the increased amount of active materials. However, a slight deviation in the group with a deposition time of 1250 s is observed, possibly due to the poor conductivity of MnO_2 materials. The charge/discharge mode with a small current imitates the situation of an integrated system under dim light or indoor light where a trace current charges the supercapacitor. In contrast, the capacitances at high current densities are similar across groups with different deposition times, as severe concentration polarization and the large iR drop from the intrinsic poor conductivity of manganese oxides become dominant factors. **Fig. S6D** clearly shows the exponential decay of areal capacitance, indicating the room for improvement, which has been attained by adjusting the interspace

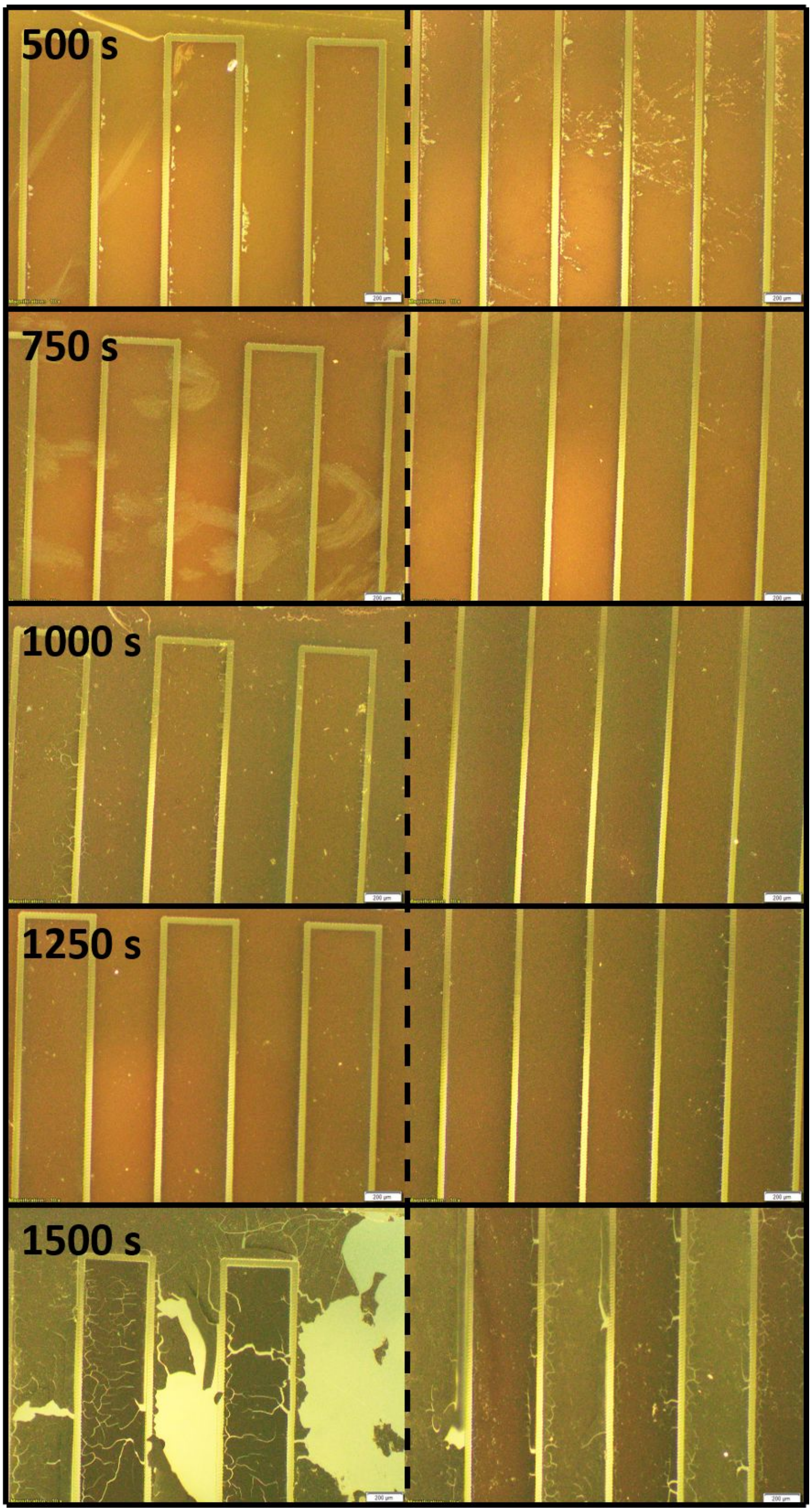

Figure S7. The Optical microscopic observations of electrodeposited manganese oxide complexes with deposition time of 500 s, 750 s, 1000 s, 1250 s.

3. PVP-graphene sensor

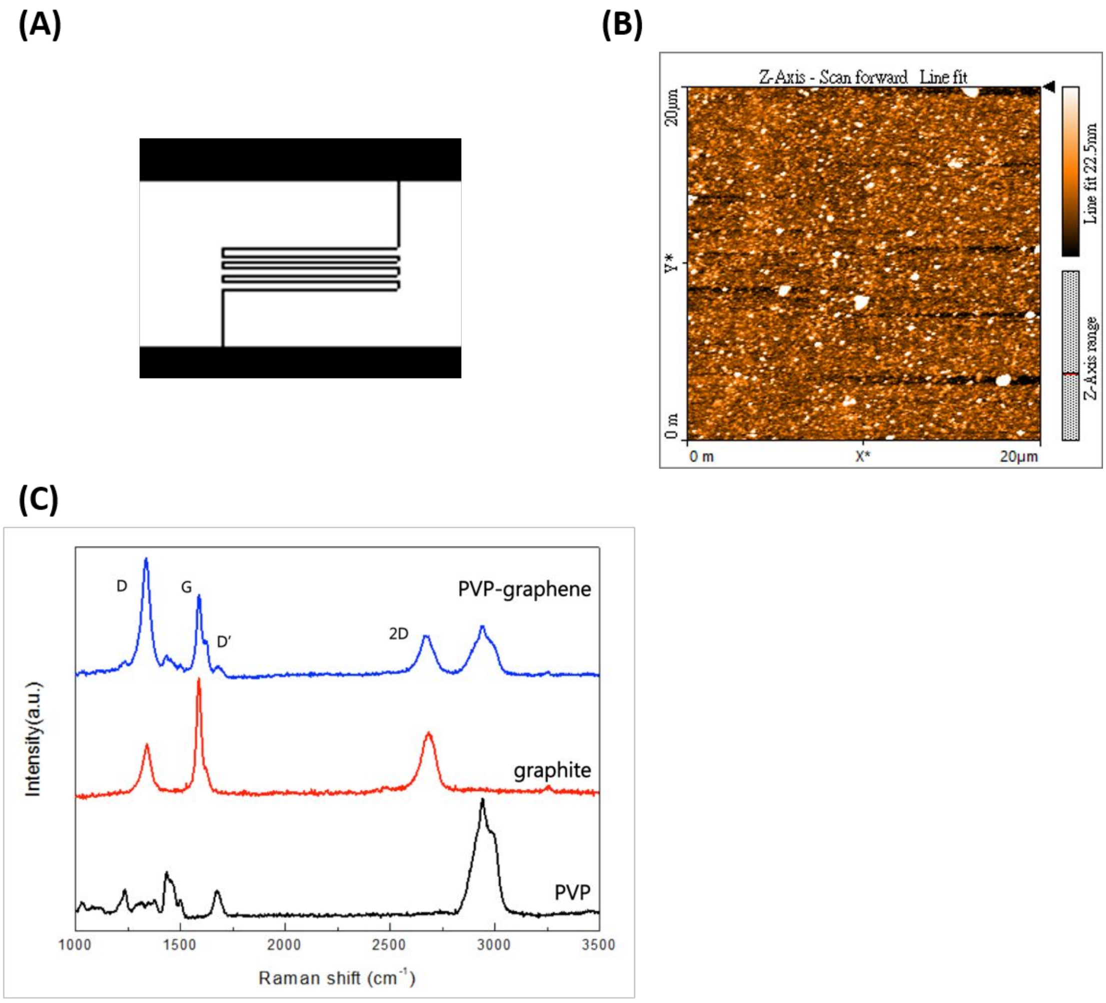

Figure S8 (A) The configuration of the sensor based on Pattern II. (B) The AFM image of PVP-graphene on the Si wafer. (C) Raman spectra of the PVP-graphene, graphite, and PVP powder.

Fig. S8A illustrates the configuration of the sensor component within the integrated devices. PVP-graphene is deposited in the insulated gap between two electrodes so that the resistance change of the PVP-graphene can be detected by measuring the current through the electrodes. In **Fig. S8B**, the surface topography of PVP-graphene on the Si wafer is presented. The morphology of PVP-graphene is well-distributed on the Si surface with a small arithmetical mean height (Sa) of 3.38 nm. Sa

implies that the PVP-graphene has about 10 layers with a van der Waals thickness of approximately 0.34 nm, which is consistent with the TEM data reported in our previous work.[1] Additionally, the Raman spectrum of PVP-graphene (**Fig. S8C**) displays a relatively broad and weak 2D band (2700 cm^{-1}), indicating the presence of few layers of PVP-graphene nanoplates, which was in good agreement with the TEM and AFM results.[1] Furthermore, a significantly higher D/G intensity ratio than that of graphite powders indicates the increased edge defects during the PVP exfoliation process.

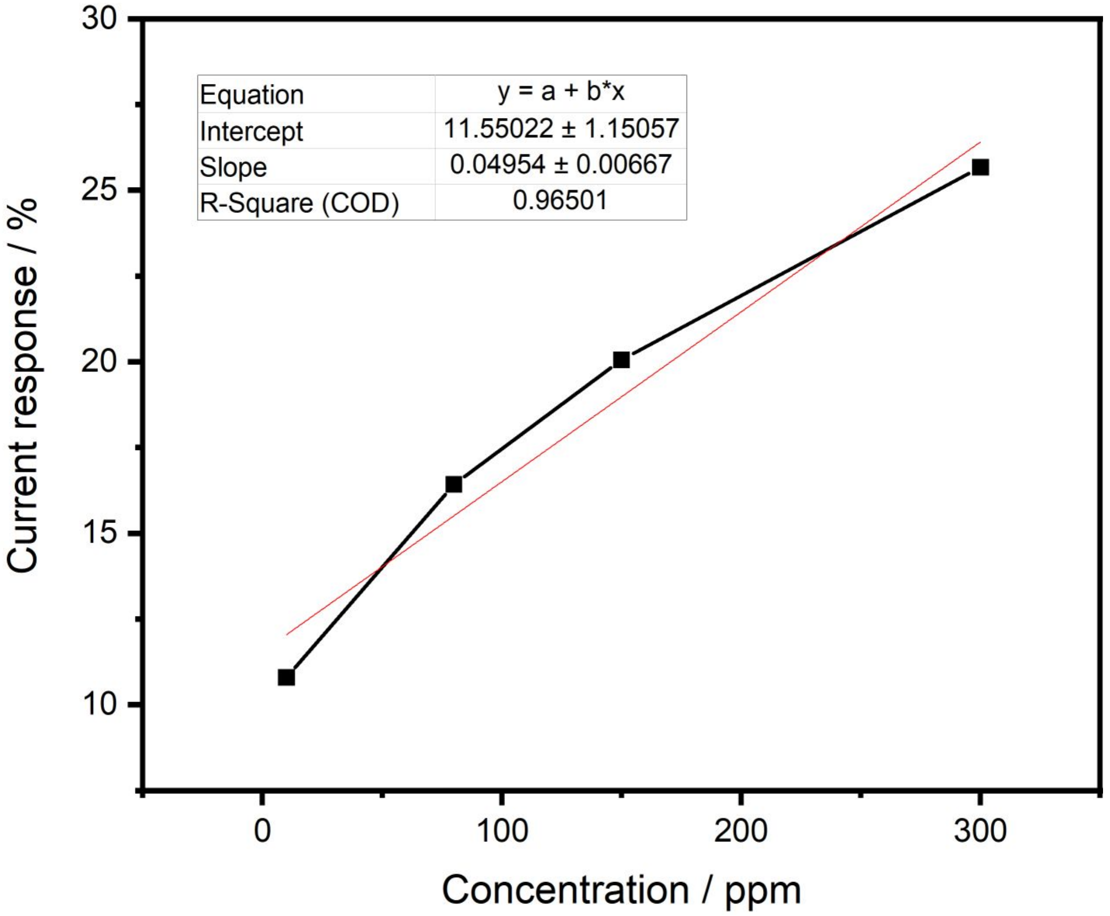

Fig. S9. The correlation between response and concentration.

4. Photo-capacitor

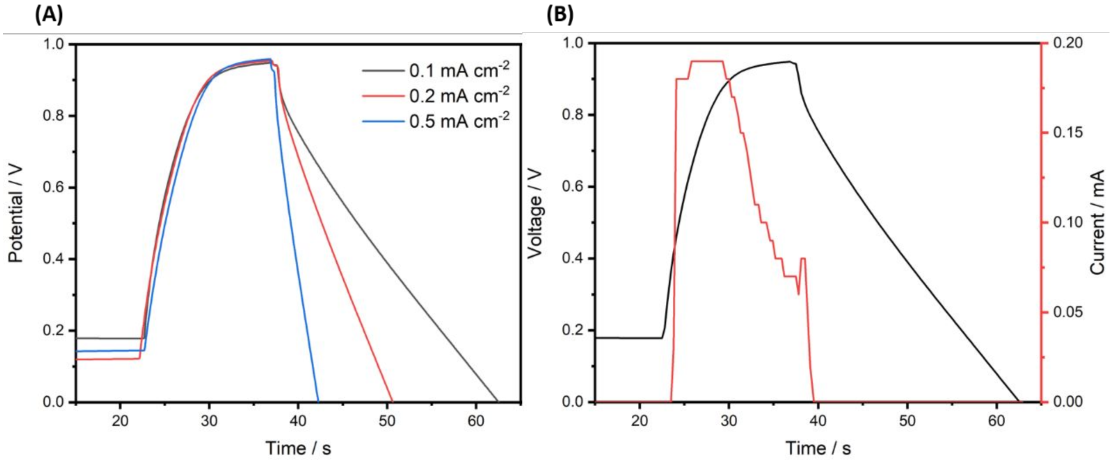

Figure S10 (A) The photo-charging and dark galvanostatic discharge of photo-capacitor at various current densities. (B) The voltage and current output of a perovskite solar cell when the photo-capacitor are photo-charged and discharged at 0.1 mA cm^{-2}.

A photo-capacitor was created by integrating a perovskite solar cell and a supercapacitor on Pattern II. The efficiency of the photo-capacitor was evaluated by charging in 15 s. From **Fig. S10A**, it can be seen that the photo-capacitor was quickly charged to the 0.96 V under illumination and galvanostatically discharged to 0 V in the dark. The discharged capacitance of the photo-capacitor at current densities of 0.1, 0.2, 0.5 mA cm^{-2} in the dark were 2.69, 2.86, 2.84 mF respectively. The comparable capacitance values indicate that the photo-capacitor can be photo-charged to a high state of charge in a short amount of time. The energy conversion and storage of photo-capacitor undergo two process: light-to-electric energy conversion (photo-charging), and electric-to-chemical-to-electric output. The overall efficiency can thus be represented by the multiplication of these two single process efficiencies ($\eta_{overall} = \eta_{L-E} \times \eta_{E-C-E}$). These efficiencies are defined based on the following formula:

(1) Light-to-electric energy conversion efficiency (η_{L-E}) of the device:

$$\eta_{L-E} = \frac{E_{out}}{E_{in}}$$

$$E_{in} = P_{in} * S_{PSC} * (t_2 - t_1)$$

$$E_{out} = \int_{t1}^{t2} I_{out}(t) V_{out}(t) dt$$

Where E_{in} is the light energy (mW s) received, and E_{out} is the electric energy output (to supercapacitor) (mW s) during photo-charging time period (t_1 to t_2). Among them, P_{in} is the incident light power density (mW cm^{-2}), S_{PSC} is the effective light area (cm^2), while I_{out} (mA) and V_{out} (V) are the output current and voltage from perovskite solar cell during light charging section.

(2) Electric-to-chemical-to-electric output efficiency (η_{E-C-E}) of the device:

$$\eta_{E-C-E} = \frac{E_d}{E_{out}}$$

$$E_d = \frac{1}{2} C * V^2$$

Where E_d is the electric energy output of supercapacitor during dark discharge, C is the capacitance (mF), and V is the voltage output (V) of the supercapacitor.

(3) Overall efficiency ($\eta_{overall}$) of the device:

$$\eta_{overall} = \eta_{L-E} \times \eta_{E-C-E} = \frac{E_d}{P_{in} \times S_{PSC} \times (t_2 - t_1)}$$

From these equations, it was calculated that at a current density of 0.1 mA cm^{-2}, the light-to-electric energy conversion efficiency (η_{L-E}), electric-to-chemical-to-electric output efficiency (η_{E-C-E}), and overall efficiency ($\eta_{overall}$) are calculated as the 0.37%, 59.65%, and 0.22%, respectively. The moderate η_{E-C-E} indicates the supercapacitor can operate and convert the energy properly, while the low η_{E-C-E} could result from another issue. To identify the key factor, the voltage output and current output of perovskite solar cell were examined (see **Fig. S10B**). It was found that the current output was quite lower than J_{sc} (i.e. 3.43 mA), which could be limited by the supercapacitor. The result suggests the mismatch in charge capacity between the

solar cell and supercapacitor will lead to a low device efficiency, and should be taken into consideration in future work.

5. PSC-MnSC-GnSR fully integrated sensing system

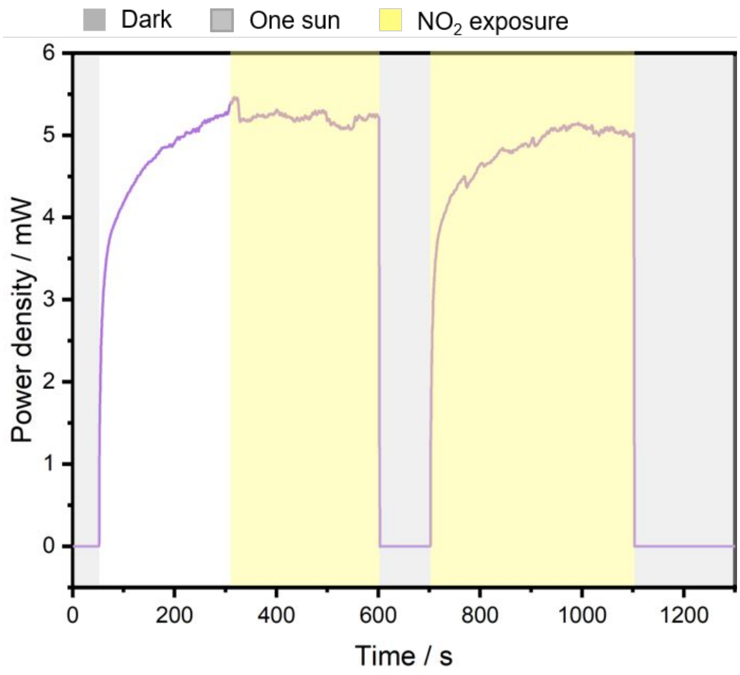

Figure S11 The power output of the solar cell in the fully integrated sensing system.

Self-powered integrated sensing system

Table S1. The sensing performance comparison of recent self-powered sensing systems.

Energy devices	Sensing objects	Response value (%)	Refs.
**Perovskite-PVs/MnO_2 MSCs**	**NO_2**	**14.8 (62.5 ppm)**	**This work**
Si-PVs/GC-MSCs	NH_3	21.4 (100 ppm)	[2]
	aniline	19.4 (100 ppm)	
Si-PVs/MSCs	ethanol	13.2 (1.54%)	[3]
	acetone	3.1 (6.07%)	
PNAI@CNT-MSCs	NO_2	~12.5 (200 ppm)	[4]
NiO/ZnO nanogenerators	H_2S	31.5 (100 ppm)	[5]
PPy-MSCs	ethanol	~13 (50 ppm)	[6]
PANI-TENG	NH_3	~3.2 (500 ppm)	[7]
Graphene-PANI-MSCs	NO_2	~12 (200 ppm)	[8]
	NH_3	~12 (200 ppm)	
CNT-MSCs	NO_2	20 (200 ppm)	[9]
rGO-MSCs	acetone	~12 (100 ppm)	[10]

PVs: photovoltaics

MSCs: micro-supercapacitors

TENG: triboelectric nanogenerator.

Reference

(1) Liu, Y. C.; Zhai, P.; Lu, M. N.; Lee, C. C.; Reddy, K. S. K.; Tingare, Y.; Yeh, C. Y.; Wei, T. C. Platinum-Free Counter Electrode Using Polymer-Capped Graphene Nanoplatelets for Cobalt (Ii)/(Iii)-Mediated Porphyrin-Sensitized Solar Cells. *Energy Technology* **2017,** *5* (5), 756-764.

(2) Shi, X.; Chang, J.; Qin, J.; Liu, H.; Zhang, X.; Ma, Y.; He, J.; Chou, X.; Feng, L.; Wu, Z.-S. Scalable Fabrication of in-Plane Microscale Self-Powered Integrated Systems for Fast-Response and Highly Selective Dual-Channel Gas Detection. *Nano Energy* **2021,** *88*, 106253.

(3) Lin, Y.; Chen, J.; Tavakoli, M. M.; Gao, Y.; Zhu, Y.; Zhang, D.; Kam, M.; He, Z.; Fan, Z. Printable Fabrication of a Fully Integrated and Self-Powered Sensor System on Plastic Substrates. *Adv. Mater.* **2019,** *31* (5), 1804285, DOI: 10.1002/adma.201804285.

(4) Yun, J.; Lim, Y.; Jang, G. N.; Kim, D.; Lee, S.-J.; Park, H.; Hong, S. Y.; Lee, G.; Zi, G.; Ha, J. S. Stretchable Patterned Graphene Gas Sensor Driven by Integrated Micro-Supercapacitor Array. *Nano Energy* **2016,** *19*, 401-414, DOI: 10.1016/j.nanoen.2015.11.023.

(5) Qu, Z.; Fu, Y.; Yu, B.; Deng, P.; Xing, L.; Xue, X. High and Fast H2s Response of Nio/Zno Nanowire Nanogenerator as a Self-Powered Gas Sensor. *Sens. Actuators B Chem.* **2016,** *222*, 78-86, DOI: 10.1016/j.snb.2015.08.058.

(6) Li, L.; Fu, C.; Lou, Z.; Chen, S.; Han, W.; Jiang, K.; Chen, D.; Shen, G. Flexible Planar Concentric Circular Micro-Supercapacitor Arrays for Wearable Gas Sensing Application. *Nano Energy* **2017,** *41*, 261-268.

(7) Cui, S.; Zheng, Y.; Zhang, T.; Wang, D.; Zhou, F.; Liu, W. Self-Powered Ammonia Nanosensor Based on the Integration of the Gas Sensor and Triboelectric Nanogenerator. *Nano Energy* **2018,** *49*, 31-39, DOI: 10.1016/j.nanoen.2018.04.033.

(8) Ye, J.; Tan, H.; Wu, S.; Ni, K.; Pan, F.; Liu, J.; Tao, Z.; Qu, Y.; Ji, H.; Simon, P.; Zhu, Y. Direct Laser Writing of Graphene Made from Chemical Vapor Deposition for Flexible, Integratable Micro-Supercapacitors with Ultrahigh Power Output. *Adv. Mater.* **2018,** *30* (27), 1801384, DOI: 10.1002/adma.201801384.

(9) Song, C.; Yun, J.; Lee, H.; Park, H.; Jeong, Y. R.; Lee, G.; Kim, M. S.; Ha, J. S. A Shape Memory High-Voltage Supercapacitor with Asymmetric Organic Electrolytes for Driving an Integrated No2 Gas Sensor. *Adv. Funct. Mater.* **2019,** *29* (24), 1901996, DOI: 10.1002/adfm.201901996.

(10) Ai, Y.; Lou, Z.; Chen, S.; Chen, D.; Wang, Z. M.; Jiang, K.; Shen, G. All Rgo-on-Pvdf-Nanofibers Based Self-Powered Electronic Skins. *Nano Energy* **2017,** *35*, 121-127, DOI: 10.1016/j.nanoen.2017.03.039.
